# Enhanced Water Sorption Performance of Polyacrylamide & Glass Fiber Paper Composites: Investigation and Comparison of Application in Desiccant Wheels

**DOI:** 10.3390/polym15183678

**Published:** 2023-09-06

**Authors:** Yimo Liu, Zhongbao Liu, Zepeng Wang, Weiming Sun

**Affiliations:** Department of Environment and Life, Beijing University of Technology, Beijing 100124, China; nxlym@emails.bjut.edu.cn (Y.L.); wangzp@emails.bjut.edu.cn (Z.W.); swimming5734@gmail.com (W.S.)

**Keywords:** polyacrylamide, aluminum fumarate, water sorption, dehumidification, desiccant wheel

## Abstract

The water sorption and desorption properties of solid adsorbent materials are crucial in rotary dehumidification systems. Metal organic frameworks (MOFs) and hydrogels are mostly at the laboratory stage due to factors like the synthesis process and yield. In this study, we utilized an eco-friendly and large-scale synthesis method to prepare polyacrylamide (PAM) hydrogels (yielding approximately 500 mL from a single polymerization). Subsequently, PAM was then coated onto glass fiber paper (GFP), which serves as a commonly employed substrate in desiccant wheels. By incorporating the hygroscopic salt LiCl and optimizing the content of each component, the water sorption performance of the composite was notably improved. The water sorption and desorption performances, as well as cycling stability, were evaluated and compared with composites containing aluminum fumarate, LiCl, and GFP (AlFum-LiCl&GFP). The results revealed that PAM-LiCl&GFP outperformed AlFum-LiCl&GFP in terms of sorption capacity throughout various relative humidity (RH) levels. It achieved a water uptake of 1.06 g·g^−1^ at 25 °C and 30% RH, corresponding to a water sorption rate coefficient K of 15.32 × 10^−4^ s^−1^. Furthermore, the lower desorption temperature (60 °C) resulting in a desorption ratio of 82.6%, along with the excellent cycling stability and effective performance as a desiccant wheel module, provide evidence for the potential application of PAM-LiCl&GFP in desiccant wheels.

## 1. Introduction

Humidity control is of great significance to human life and plays a crucial role in various industrial processes, especially in precision electronics manufacturing [1]. Currently, commonly used dehumidification methods include condensation dehumidification [2], membrane dehumidification [3], and adsorption–absorption dehumidification [4]. Among these methods, adsorption dehumidification has garnered significant attention due to its capability to utilize low-grade waste heat sources, such as solar energy and industrial waste heat [5]. Rotary dehumidification, as a type of adsorption dehumidification, offers continuous operation and is widely used in practical applications. Furthermore, rotary dehumidification offers benefits, such as a low dew point temperature and a large adsorption area [6].

The choice of adsorbents plays a critical role in the rotary dehumidification system, greatly impacting both the operational temperature range and dehumidification efficiency. Presently, silica gel and zeolite are commonly utilized materials for the desiccant wheel in this system. However, zeolite requires a high regeneration temperature, frequently surpassing 100 °C [7], leading to increased energy consumption. On the other hand, even though silica gel has a lower regeneration temperature, its dehumidification performance at low-dew-point temperatures is still unsatisfactory [8]. Consequently, it is imperative to utilize a desiccant that exhibits outstanding dehumidification performance and operates at a low desorption temperature, suitable for application in rotary dehumidification systems. Among them, polymers are potential choices for quality adsorbents.

Metal organic frameworks (MOFs) are a relatively new type of polymer adsorbent that exhibit high porosity and hydrophilicity due to rational composition and pore structure design [9,10]. Several MOFs, including MOF-801 [11], CPO-27Ni [12], MIL-101(Cr) [13], and MIL-100(Fe) [14], have shown promising potential for applications, such as adsorption cooling and air water harvesting. However, their absorption capacity is mostly limited to approximately 0.5 g·g^−1^ [15]. The incorporation of hygroscopic salts in MOFs enhances the dehumidification performance even further [16,17]. An example is CaCl_2_@MOF-808, which demonstrates a water uptake of 0.56 g·g^−1^ at 25 °C and 30% relative humidity (RH) [18]. Chen et al. [19] conducted a study where they combined MIL-101 (Cr) with ceramic fiber paper (CFP) using a coating method and found that the resulting water uptake was 100 mg·g^−1^ higher than that of commercially available silica gel&CFP at 70% RH. However, the complex synthesis and low yield hinder the application of MOF in rotary dehumidification, despite its noteworthy adsorption performance and favorable desorption temperature.

Covalent organic frameworks (COFs) are crystalline polymers with modular tunability and well-structured pores [20], making them highly significant in the field of adsorption. Furthermore, COFs, such as COF-432 [21], with a maximum water uptake of 0.3 g·g^−1^ at a low relative humidity, and COF-480-hydrazide [22], with a stable uptake of around 0.45 g·g^−1^, have demonstrated their potential in water harvesting. However, the COF-480-hydrazide exhibits certain hysteresis in water adsorption [21].

Recently, hydrogel polymers have garnered significant research attention in water sorption due to their excellent hydrophilicity and low desorption temperature [23,24,25,26]. To enhance the hygroscopicity of hydrogels, it is often necessary to incorporate hygroscopic factors, like inorganic salts, which are highly responsive to water vapor. These composite materials are commonly referred to as super-adsorbent hydrogels (SAHs) [27,28,29]. For instance, P(AA-co-AM) super-porous hydrogel composites prepared using gas blowing and foaming techniques can achieve a maximum water uptake of 1.03 g·g^−1^ [30]. Moreover, SAMG hydrogels synthesized by infiltrating polypyrrole chloride into poly N-isopropylacrylamide, as investigated by Zhao et al. [31], exhibit a water uptake of up to 3.4 g·g^−1^ at 25 °C and 60% RH relative humidity. Furthermore, SMAG can be regenerated at desorption temperatures of 50 °C. Li et al. [32] conducted air–water extraction experiments using hydrogels based on carbon nanotubes (CNTs), polyacrylamide (PAM), and CaCl_2_. The water uptake of PAM was 0.32 g·g^−1^, while, upon the introduction of CaCl_2_, the absorption amount elevated to a satisfactory level of 2.03 g·g^−1^. Lu et al. [33] utilized 1 g PAM-LiCl to develop an air-water extraction prototype, achieving a water yield of up to 0.5 g·g^−1^ per cycle at 20% RH. Besides possessing strong hygroscopic properties, the desorption heat—stemming from the water’s interaction, captured by the deliquescence of hygroscopic factor LiCl, with the polymer hydrogel network (PAM-LiCl)—was considerably lower than conventional salt adsorbents. In scenarios where SAHs capture airborne water vapor, the vapor is primarily taken in through spontaneous chemisorption via hygroscopic salts, and the crosslinked SAHs polymer matrix prevents leakage of deliquescent salts, retaining the water in a swollen gel structure. Consequently, SAH swells in volume as the water uptake intensifies [33].

**Figure 1:**
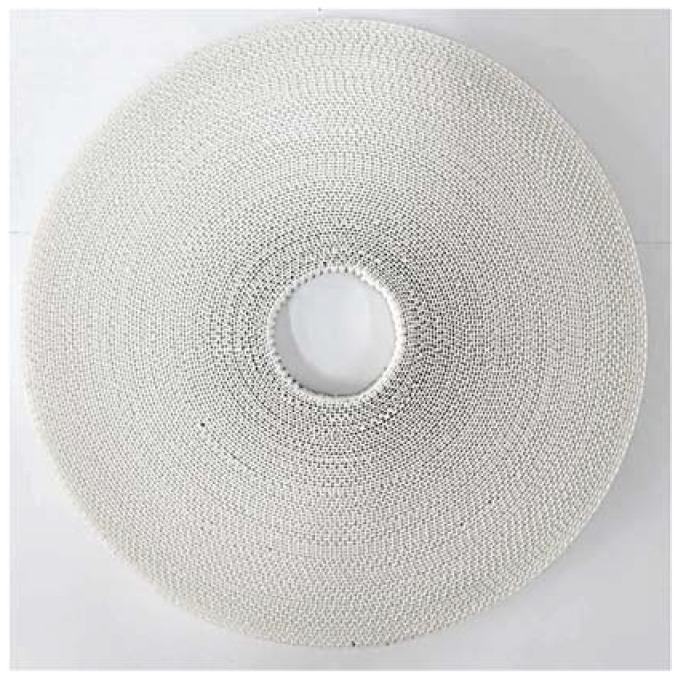
Photograph of the substrate of desiccant wheels.

Desiccants, specifically SAHs with outstanding properties, have been documented in solid dehumidification [34,35]. Yet, to our best knowledge, there is a limited number of reports on their applications in rotary dehumidification. The majority of recent studies on desiccant wheels emphasize MOFs, like MIL-101 (Cr) [19], AlFum [36], and MIL-100 (Fe) [37], as well as mesoporous nano-adsorbent SiO_2_ [38] and polyacrylic acid [39,40]. The integration of MOFs with hygroscopic salts introduces a risk of salt leakage, stemming from its intrinsic pore space limitations [18], and many reports on SAHs pertain to small-scale, laboratory settings. Conversely, the volume expansion that accompanies SAHs’ superior water sorption properties presents challenges in rotary dehumidification applications. This expansion results in the clogging of the pores in their primary carrier: a cylindrical honeycomb skeleton constructed from glass fiber paper (GFP) or CFP, often referred to as the rotor. This clogging, in turn, impairs the mass transfer and dehumidification performance (as depicted in Figure 1).

Consequently, this study details the synthesis of PAM using the green macro method and combines it with GFP—a standard substrate for rotary dehumidification—via the impregnation method. Upon the addition of the hygroscopic salt LiCl, we achieved an optimized PAM-LiCl&GFP composition. Dehumidification and desorption performances were assessed using the univariate method under varied conditions. This was contrasted with the performance of AlFum-LiCl&GFP, an earlier composite we developed for rotary dehumidification [41]. We examined the cycling stability of PAM-LiCl&GFP under ideal conditions. Subsequently, the desiccant wheel module using PAM-LiCl&GFP was assembled and evaluated through initial experiments.

## 2. Experimental Materials and Method

### 2.1. Materials

We employed free-radical polymerization to crosslink acrylamide monomers, facilitating PAM preparation. Acrylamide (AM, Analytical Reagent, AR) was purchased from the Shanghai Macklin Biochemical Co., Shanghai, China. Additionally, the crosslinking agent N’,N’-methylenebis (MBA, Chemically Pure, CP), the catalyst N,N,N’,N’-tetramethylethylenediamine (TEMED, AR), the initiator potassium persulfate (KPS AR), and the hygroscopic salt lithium chloride (LiCl, AR) were utilized. Aforementioned materials were obtained from the Aladdin Industrial Company, Shanghai, China. and were used without additional purification.

### 2.2. Preparation of PAM

AM was dissolved in deionized water using magnetic stirring, yielding a 10% AM solution. Nitrogen was introduced into the AM solution for 10 min, removing dissolved oxygen that could interfere with free-radical polymerization by quenching active radicals. Then, 0.8 mL of 3.8 mg·mL^−1^ MBA solution, 0.4 mL of 50 mg·mL^−1^ KPS solution, and 50 µL of TEMED were incorporated into every 10 mL of the previously prepared AM solution, utilizing ultrasonic vibrations. Sonication persisted until AM began polymerizing, as evidenced by the solution becoming gelatinous and non-flowing. The container was set aside overnight, rinsed 2–3 times with deionized water, and then dried in a convection oven at 80 °C to yield water-free PAM gels. The polymerization process for the AM solution is isometrically scalable. In this study, we polymerized around 500 mL of AM solution in a single batch, producing 500 mL of watery PAM hydrogels. Using multiple containers allows for the production of greater quantities of PAM hydrogels from a singular batch.

### 2.3. Preparation of Adsorbent&GFP

GFP (purchased from Wuxi Desert Dehumidification Equipment Factory, Wuxi, China) was cut into 50 × 50 mm squares, and the mass *m*_GFP_ was measured immediately after drying in a convection oven at 80 °C for three hours.

The dried PAM hydrogel was processed into powder and then introduced into deionized water. The mixture underwent magnetic stirring for 30 min, resulting in PAM solutions with concentrations of 2%, 4%, and 6%. Through grinding, we aimed to ensure a more uniform dispersion of PAM in the deionized water. GFP underwent immersion in the PAM solution for 2 min and were then extracted and left hanging for 10 min to shed any excess solution. They were then subjected to drying in a convection oven at 80 °C for one hour, with the resulting mass denoted as *m*_b_, g.

**Figure 2:**
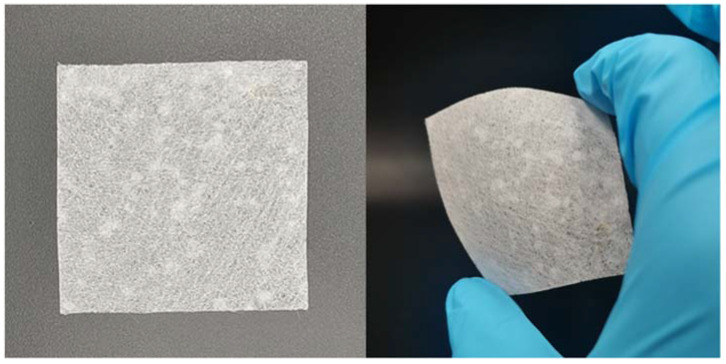
Photograph of the PAM-LiCl&GFP.

Solutions of LiCl with concentrations ranging from 10% to 30% were formulated. The dried PAM&GFP samples were soaked in these solutions for 2 min before being extracted and left hanging for 10 min. They were later dried in a convection oven at 80 °C for one hour. Subsequent to that, surplus LiCl precipitate on the GFP sheets was delicately removed with a cotton ball soaked in anhydrous ethanol. The samples underwent an added 10 min drying period and were then weighed to establish the mass of the resultant PAM-LiCl&GFP, denoted as *m*_c_, g. Adsorbents and desiccant wheel modules were crafted following the aforementioned method. A visual representation of the PAM-LiCl&GFP can be found in Figure 2.

Sample designations for PAM-LiCl&GFP ranged from 2 + 10% to 6 + 30%, reflecting the varied concentrations of GFP impregnated with PAM and LiCl. The naming convention dictates that the PAM solution concentration precedes that of the LiCl solution. Quantities of PAM (represented as *m*_PAM_, g) and LiCl (notated as *m*_LiCl_, g) were deduced utilizing Equations (1) and (2), respectively.

(1) $$m_{PAM}=m_{b}-m_{GFP},$$

(2) $$m_{LiCl}=m_{c}-m_{b}.$$

The method and essential materials for crafting AlFum-LiCl&GFP are outlined in our previous work [41] and will not be recapped in this paper.

### 2.4. Characterization

Fourier-transform infrared (FTIR) spectra of the samples were recorded by a Thermo Scientific Nicolet iS20 instrument (Waltham, MA, USA) at room temperature. The surface morphology and elemental distribution mapping of the samples were examined with a scanning electron microscope (SEM, Czech TESCAN MIRA LMS) and energy dispersive spectroscopy (EDS), respectively. Differential scanning calorimetry (DSC) was conducted using a TA Q2000, DSC2500 instrument (New Castle, DE, USA) to determine the thermal behavior of the PAM-LiCl&GFP. Measurements proceeded in a nitrogen atmosphere, with a heating pace of 5 °C∙min^−1^. Water sorption and desorption tests for the samples were performed in an MT/150 L chamber, maintaining constant temperature and humidity. This chamber allows adjustments in dry bulb temperature (DB) and RH as needed, with a consistent airflow at 2.15 m∙s^−1^ ensuring stable ambient conditions. The specific surface areas were calculated by the Brunauer–Emmett–Teller (BET) method via Micromeritics ASAP 2460 (Norcross, GA, USA).

### 2.5. Dehumidification and Desorption Performance Test

The sample is oven dried at 80 °C for an hour to ensure complete removal of all adsorbed water. Next, the initial mass is determined and noted as *m*_a_, g. Once the constant temperature and humidity chamber reach a stable ambient condition, the sample is introduced, and the sample’s mass (*m*_c_, g) is monitored and logged periodically. The water uptake *w* (g·g^−1^) per gram of the test sample is calculated by dividing the difference between *m*_c_ and *m*_a_ by the initial mass *m*_a_:

(3) $$w=\frac{m_{c}-m_{a}}{m_{a}}.$$

When water sorption reaches saturation—signified by the sample’s mass changing by less than 0.01 g three times consecutively—the weight is noted as the equilibrium water sorption mass, *m*_e_. Desorption tests were similarly conducted in the constant-temperature and -humidity chamber. Throughout this process, the mass of the sample is periodically assessed and logged as *m*_c_. The desorption ratio, *r*, of the sample is calculated using Equation (4):

(4) $$r=\frac{m_{e}-m_{c}}{m_{e}-m_{a}}\times 100\%.$$

The water uptake *w* per gram of adsorbent and the desorption ratio *r* account for both the self-weight of the GFP substrate and the weight of the adsorbents. This consideration is crucial for real-world engineering applications, especially in desiccant wheel dehumidification systems, providing deeper insights than merely a material-centric perspective.

### 2.6. Uncertainty Analysis

Errors were accounted for in the analysis of the experimental results. The primary sources of error are the measurement techniques and the precision of the equipment used. The main parameter assessed was mass, measured with an electronic balance (ME802E) having a precision of 0.0001 g within a range of 0–100 g. Using the error transfer equation, the variations in the water uptake *w* and the desorption ratio *r* under varying conditions are between 0.11% and 3.99%, which is considered acceptable.

The loading of the adsorbent on GFP is inherently inconsistent due to the small-batch production in the laboratory and the viscous quality of the PAM solution. In this regard, the loading differences of various PAM concentrations on GFP are quantified using the standard deviation (SD), and the findings are showcased in Section 3.2.1, where the optimization of PAM content is highlighted.

### 2.7. Water Sorption Kinetic

To elucidate the dynamic water sorption properties of adsorbent&GFP, the rate coefficients of sorption for the samples were analyzed using the linear driving force model (LDF), represented by Equation (5) [42]:

(5) $$\frac{dw}{dt}=K(w_{eq}-w_{t}).$$

where *t* represents time in s^−1^; *dw*/*dt* signifies the water sorption rate; *w*_t_ characterizes the dynamic water uptake in g·g^−1^; and *w*_eq_ stands for the equilibrium water uptake in g·g^−1^. Integral transformations applied to both sides of the aforementioned equations yield:

(6) $$\frac{w_{t}-w_{a}}{w_{eq}-w_{a}}=1-e^{-Kt}.$$

where *w*_a_ denotes the initial water uptake in g·g^−1^. Within the water sorption process, this value is customarily set to zero, as the test sample undergoes regeneration prior to the sorption assay. Upon reconfiguring Equation (6) and implementing the natural logarithm, Equation (7) results:

(7) $$-ln\;(1-\frac{w_{t}}{w_{eq}})\;=\;Kt.$$

## 3. Results and Discussion

### 3.1. Characterization of the Samples

#### 3.1.1. FTIR Analysis

**Figure 3:**
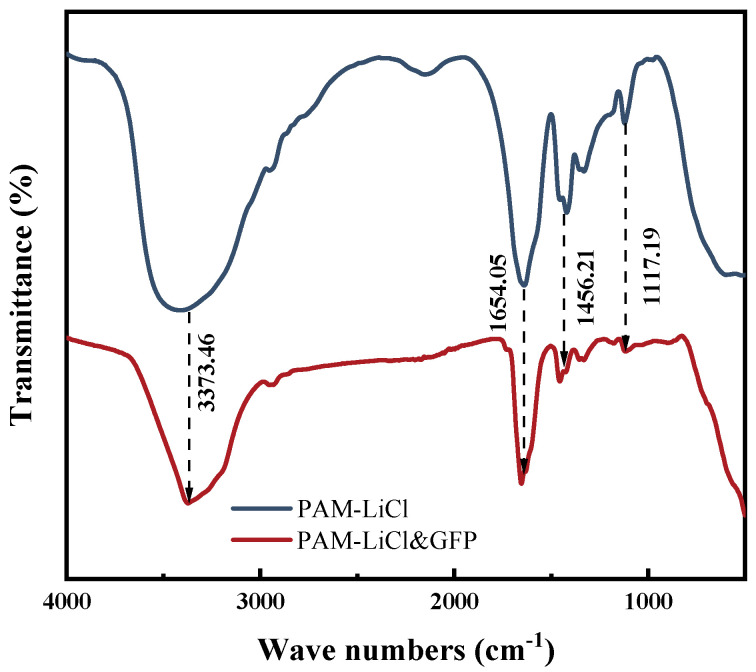
FTIR spectra of PAM-LiCl and PAM-LiCl&GFP.

To examine the chemical functional group structure of the PAM-LiCl hydrogel and its successful integration on GFP, FTIR analysis was performed, as shown in Figure 3. The blue curve illustrates the infrared spectra of PAM-LiCl, with the feature at 3373.46 cm^−1^ associated with the stretching vibration of -NH_2_, the peak at 1654.05 cm^−1^ represents the characteristic absorption of the carbonyl group (C=O stretching vibration), the peak at 1456.21 cm^−1^ is related to the characteristic absorption of methylene deformation, and the peak at 1117.19 cm^−1^ is tied to the in-plane oscillatory vibrations of -NH_2_ in the amide molecule. When compared with the standard infrared profile, it is revealed that these characteristic absorption peaks of PAM-LiCl, synthesized by free-radical polymerization, align closely with the standard profile of PAM. The red curve shows the infrared spectra of PAM-LiCl&GFP; all the distinctive peaks of PAM-LiCl are evident in the spectra, validating the successful integration of the PAM-LiCl onto GFP.

#### 3.1.2. SEM and EDS Analysis

**Figure 4:**
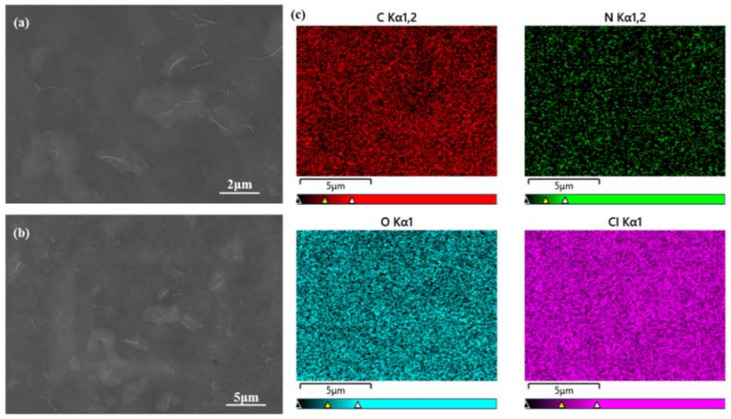
SEM and EDS images of PAM-LiCl&GFP. (**a**) SEM with a scale bar of 2 μm, (**b**) SEM with a scale bar of 5 μm, (**c**) EDS images.

Figure 4 presents the SEM and EDS micrographs of PAM-LiCl&GFP. Upon examination of Figure 4a,b, one discerns that PAM is uniformly loaded onto GFP, albeit with sporadic folds; yet, a crystalline structure is conspicuously absent. The aforementioned folds denote fine glass fibers of GFP, portions of which remain unencapsulated by PAM. The lack of crystallinity alludes to a well-dispersed LiCl solution embedded within the PAM hydrogel matrix. EDS micrographs manifest a homogeneous distribution of the elemental constituents C, N, O, and Cl, underscoring that the dispersion of LiCl remains homogeneous and is invariant to the drying procedure.

#### 3.1.3. DSC Analysis

**Figure 5:**
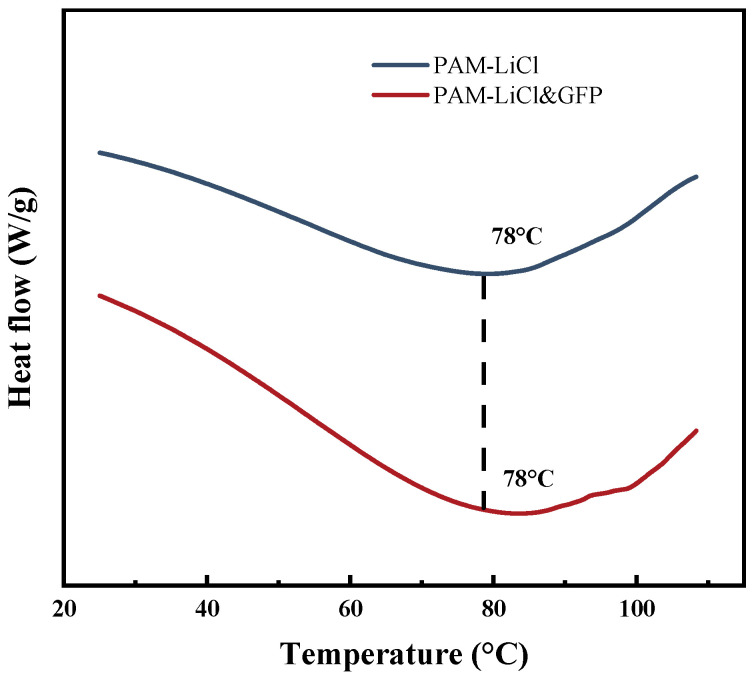
DSC thermograms showing the heat flow of PAM-LiCl and PAM-LiCl&GFP.

PAM-LiCl acts as a temperature-sensitive SAH. As evidenced in Figure 5, the DSC curve demonstrates a heat absorption peak for PAM-LiCl at 78 °C during a temperature ramp from 25 °C to 110 °C. Concomitantly, its weight loss rate reaches a zenith at 78 °C. The behavior of PAM-LiCl&GFP closely parallels that of PAM-LiCl. This implies that, in real-world applications, a desiccant wheel imbued with PAM-LiCl can be efficaciously regenerated utilizing a heat source below 100 °C.

### 3.2. Water Sorption Performance Analysis

#### 3.2.1. Optimization of PAM and LiCl Contents

**Figure 6:**
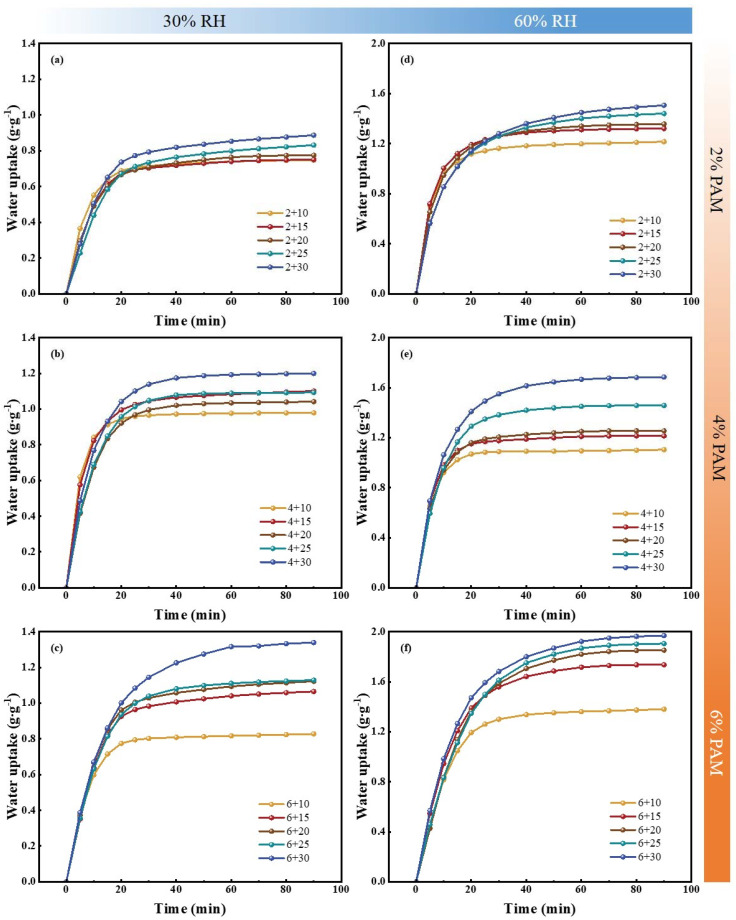
Dehumidification experiment results of different contents of PAM and LiCl. (**a**) 2% PAM + LiCl at 25 °C, 30% RH; (**b**) 4% PAM + LiCl at 25 °C, 30% RH; (**c**) 6% PAM + LiCl at 25 °C, 30% RH; (**d**) 2% PAM + LiCl at 25 °C, 60% RH; (**e**) 4% PAM + LiCl at 25 °C, 30% RH; (**f**) 6% PAM + LiCl at 25 °C, 30% RH.

Samples of PAM-LiCl&GFP, with distinct PAM and LiCl concentrations, were fabricated for dehumidification experiments at 25 °C under 30% RH and 60% RH. The objective aimed at determining the optimal ratio of PAM to LiCl. Figure 6 details the experimental outcomes. Specifically, Figure 6a illustrates the outcomes of GFP impregnated with 2% PAM, paired with different LiCl ratios, and tested at 25 °C and 30% RH. Figure 6b depicts results for 4% PAM, while Figure 6c does so for 6% PAM. Figure 6d–f showcase dehumidification outcomes for their corresponding materials at 25 °C and 60% RH. Additionally, Table 1 enumerates the masses of components across varying PAM-LiCl&GFP ratios. The error in the *m*_b_—the mass of GFP at varied PAM concentrations—was quantified using standard deviation. Twenty samples from each of the three concentrations served as the basis for statistical evaluation.

Figure 6 demonstrates that the water uptake (*w*, g·g^−1^) of the adsorbent is governed by the interplay between the PAM and LiCl concentrations. For instance, samples saturated with 30% LiCl exhibit a progressive rise in *w*_eq_ as the PAM content escalates. At 25 °C, 30% RH, the *w*_eq_ for samples with 2 + 30, 4 + 30, and 6 + 30 compositions is 0.88 g·g^−1^, 1.19 g·g^−1^, and 1.39 g·g^−1^, respectively, denoting increases of 135% and 157%. This phenomenon can be attributed to the initial trapping of water vapor by the hygroscopic salt LiCl during the PAM-LiCl water sorption process, which then liquifies through deliquescence. Subsequently, the hydrophilic polymer chains of PAM-LiCl engage with the liquid water, retaining water molecules within the engorged hydrogel matrix as bound water, weakly bound water, and free water. In this scenario, PAM functions as a reservoir, adeptly conserving the liquid water post-LiCl deliquescence. However, increasing PAM content is not necessarily advantageous, primarily for two reasons. First, in contrast to porous adsorbents where water is retained within pores, in systems with PAM-LiCl, increased water uptake results in volume expansion. Consequently, the volumetric expansion resulting from the saturation of excessive PAM might surpass the dimensions of the honeycomb pores in the desiccant wheels matrix. Secondly, as elucidated in Table 1, the mass’s standard deviation (*m*_b_) for the curated PAM&GFP stands at ±0.01 g, which translates to roughly 8% of the aggregate mass. This discrepancy can be ascribed to the pronounced viscosity of the 6% PAM solution, which poses difficulties in securing a homogeneous GFP load. Given these considerations, the PAM concentration ought not to surpass 6%.

As observed in Figure 6, there is a clear correlation between increased LiCl content and enhanced water uptake. However, practically speaking, a heightened LiCl content does not always equate to superior outcomes. The primary concern lies in situations with elevated salt content; if PAM is not sufficient to retain the deliquescent liquid water, there is a potential for salt leakage, leading to corrosion of equipment during operational use. As an example, the 6 + 30 sample showcased no salt leakage during water sorption at 25 °C and 30% RH, maintaining a water uptake of 1.33 g·g^−1^. Yet, upon increasing the relative humidity to 60% RH, salt leakage manifested prior to attaining a water uptake of 1.96 g·g^−1^. As 6% PAM loaded on GFP was unable to hold more water uptake, a reduction in the LiCl content was essential to match this ambient condition. Nonetheless, from an engineering viewpoint, the 6 + 30 sample is still crucial for low-dewpoint dehumidification. Moreover, at 25 °C, 60% RH, samples with 6 + 20 and a decreased salt content exhibited no salt leakage. The other factor is that while a higher LiCl content boosts the *w*_eq_ of the samples, it also impacts the water sorption rate somewhat. For example, at 25 °C, 60% RH, the 6 + 15 sample reached 80% of its *w*_eq_ at a rate of 69.5 mg·g^−1^∙min^−1^, whereas under the same conditions, the 6 + 20 sample showed a sorption rate of just 57.6 mg·g^−1^∙min^−1^. This outcome is probably because the agglomeration of excess LiCl will induce aggregation, impeding the exposure of active sites and leading to a reduced contact area with air, thus causing lowered sorption kinetics.

**Figure 7:**
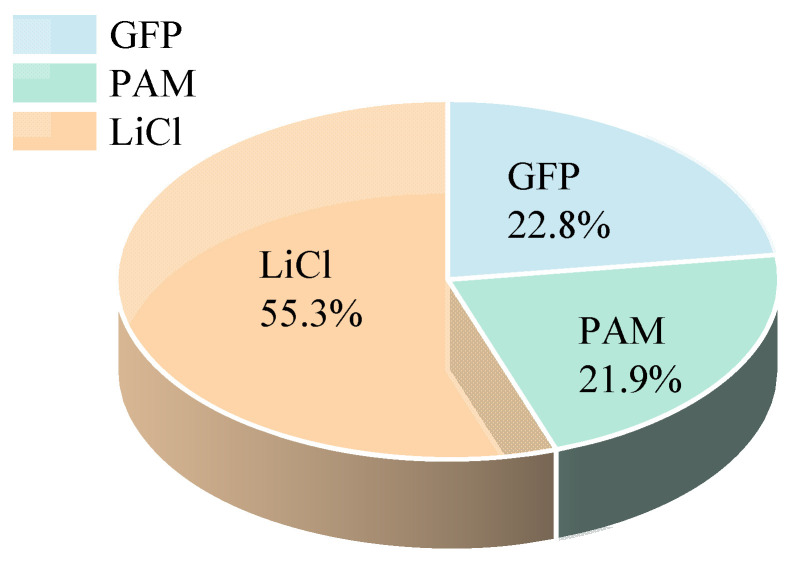
Mass ratio of each component of optimized PAM-LiCl&GFP.

Given all these factors, sample 6 + 15 was chosen as the central subject for the following discussion. The sample 6 + 15 achieved a *w*_eq_ of 1.06 g·g^−1^ at 25 °C and 30% RH, and 1.73 g·g^−1^ at 60% RH, showcasing a significant water sorption capability. Moreover, there was no evidence of salt leakage at 80% RH. As depicted in Figure 7, GFP and PAM had mass ratios of 22.8% and 21.9%, respectively, with LiCl’s mass ratio standing at 55.3%.

#### 3.2.2. Water Sorption Performance of Different Adsorbents & GFP

**Figure 8:**
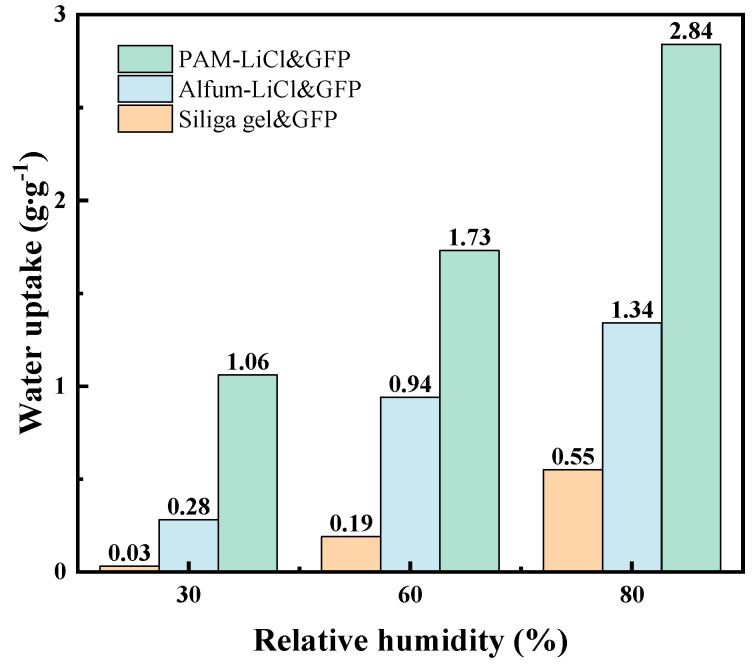
Dehumidification capacity of adsorbent&GFP.

The water uptake of different adsorbents combined with GFP was assessed by contrasting the *w*_eq_ of GFP composites, namely, silica gel, AlFum-LiCl, and PAM-LiCl, across conditions of 25 °C, 30%, 60%, and 80% RH. The results of this comparison are displayed in Figure 8. In the prior research, the water uptake of AlFum-LiCl&GFP overlooked the intrinsic weight of GFP (100 × 100 mm, 0.24 g) and only considered the water uptake of the adsorbents. To fully gauge its potential for use in desiccant wheels, the inherent weight of GFP, an essential material in wheel production, needs to be factored in and evaluated in tandem with upcoming discussion results. Figure 8 definitively indicates that PAM-LiCl&GFP delivers an exceptional water sorption result at low RH. At 30% RH, SG&GFP shows negligible sorption, registering a water uptake of just 0.03 g·g^−1^, while AlFum&GFP presents a modest *w* of 0.28 g·g^−1^. Conversely, PAM-LiCl&GFP attains an impressive 1.06 g·g^−1^, which is 3.7 times that of AlFum&GFP. Additionally, at higher RHs, specifically, 60% and 80%, PAM-LiCl&GFP displays a distinct advantage, posting water uptake of 1.73 g·g^−1^ and 2.84 g·g^−1^, respectively. This is primarily because the crosslinked network of PAM-LiCl swiftly takes in water via volume expansion, in contrast to porous adsorbents constrained by pore space limits. Nevertheless, it is vital to factor in the application scenario to avert deliquescence and clogging of pores in the desiccant wheel, as deliberated and fine-tuned in the preceding section.

**Figure 9:**
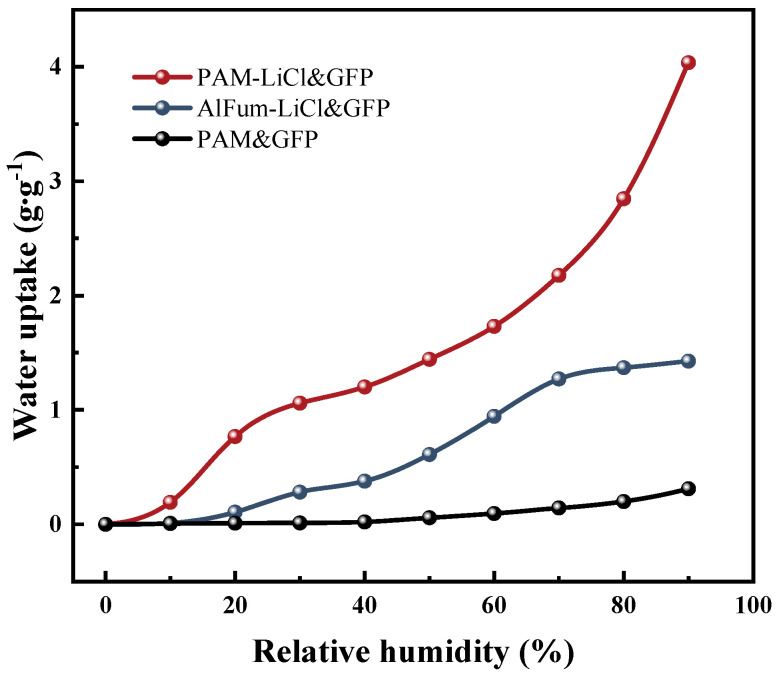
Water sorption isotherms of PAM&GFP, PAM-LiCl&GFP, and AlFum-LiCl&GFP at 25 °C.

The water sorption isotherms of PAM&GFP, PAM-LiCl&GFP, and AlFum-LiCl&GFP at 25 °C are depicted in Figure 9, with the data consolidated in Table 2 for comparison. It is evident that the non-hygroscopic salt-loaded PAM possesses negligible water sorption capacity in the low humidity range, attributed to the absence of hygroscopic sites. At high RH, there is a significant enhancement, yet it stands at only 0.31 g·g^−1^. The water vapor adsorption process of AlFum-LiCl&GFP can be bifurcated: from 10% ≤ RH < 70%, the water uptake of AlFum-LiCl&GFP rises continuously; between 70% ≤ RH ≤ 90%, it nears saturation with minimal variation. AlFum-LiCl&GFP involves both physical and chemical adsorption. At lower relative humidity, water molecules experience monomolecular layer chemical adsorption on the surface of LiCl. As relative humidity rises, water molecules are physically adsorbed within the pores of AlFum. Coupled with chemical adsorption on LiCl’s surface, this forms a multimolecular layer, obstructing further adsorption sites. Another key factor is the limited pore structure of AlFum, a type of solid adsorbent. Consequently, its water uptake tends to approach a limit.

On the other hand, the water sorption process of PAM-LiCl&GFP is predominantly driven by the hygroscopic nature of LiCl, which spontaneously captures airborne water vapor via chemisorption. The PAM-LiCl hydrogel network not just accelerates water absorption through swift swelling, but also retains the captured water, ensuring it does not leak. The diminutive BET surface area, being 8.01 m^2^·g^−1^ for PAM&GFP and 4.99 m^2^·g^−1^ for PAM-LiCl&GFP, indicates minimal physical adsorption. This correlation results in a near-linear ascent in the water uptake of PAM-LiCl&GFP as RH increases. Commencing at 10% RH and owing to LiCl’s deliquescence, the dehumidification capacity surges swiftly from 0.19 g·g^−1^ to 1.06 g·g^−1^ by 30% RH. This performance surpassed that of AlFum-LiCl&GFP in terms of equilibrium water uptake.

#### 3.2.3. Dynamic Water Sorption Performance of Adsorbent&GFP

**Figure 10:**
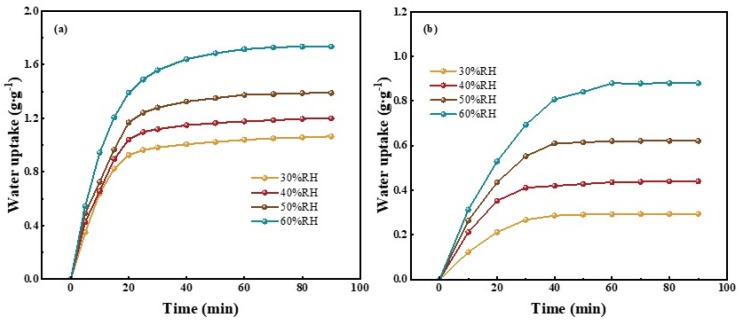
Dynamic water sorption performance at 25 °C and different RHs. (**a**) PAM-LiCl&GFP, (**b**) AlFum-LiCl&GFP.

Figure 10a,b illustrate the dynamic water sorption curves of PAM-LiCl&GFP and AlFum-LiCl&GFP at 25 °C under different relative humidity conditions. The figures show that the water uptake of adsorbent&GFP amplifies with increasing relative humidity. This can be explained by Dalton’s law of partial pressures, where a rise in relative humidity boosts the partial pressure of water vapor, thereby enhancing the water sorption process. The water sorption rate of the adsorbents declines as the sorption process advances. This occurs because the interior of the adsorbent becomes saturated with water molecules, resulting in a steady decline in the partial pressure difference between the adsorbent and the airborne water vapor. At all RH levels, PAM-LiCl&GFP attains 80% or more of its *w*_eq_ in just 20 min. However, reaching equilibrium at high RH, specifically 60% RH, requires 90 min. This trend is mainly due to the higher amount of water sorption of PAM-LiCl&GFP. Regarding the water sorption rate, PAM-LiCl&GFP registers a rate of 11.77 mg·g^−1^∙min^−1^ at 30% RH and 19.22 mg·g^−1^∙min^−1^ at 60% RH after 90 min of sorption. Conversely, AlFum-LiCl&GFP achieves a water sorption rate of 3.26 mg·g^−1^∙min^−1^ at 30% RH and 9.77 mg·g^−1^∙min^−1^ at 60% RH after 90 min of sorption. Compared to AlFum-LiCl&GFP, PAM-LiCl&GFP exhibits a higher water sorption rate.

#### 3.2.4. Water Sorption Kinetic Analysis

**Figure 11:**
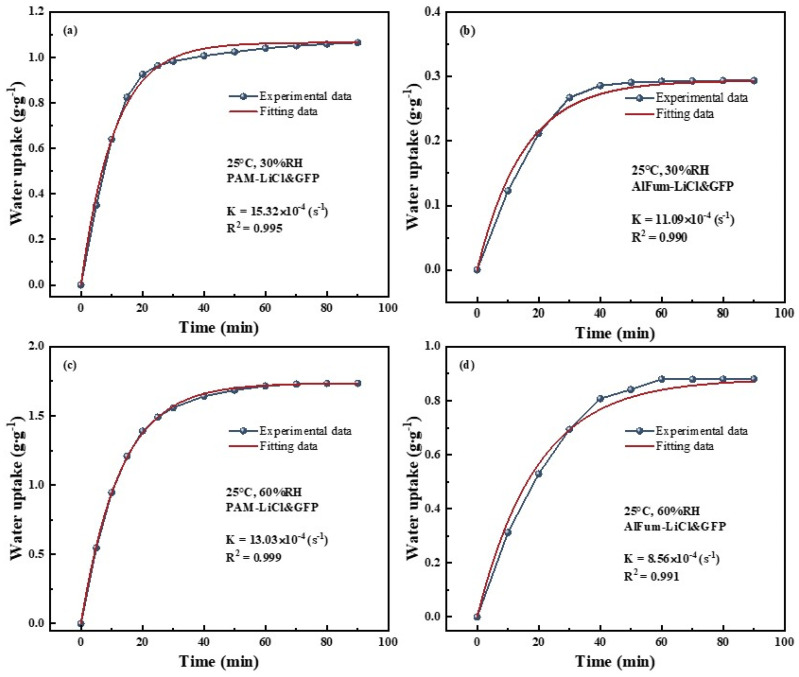
LDF fitting results for adsorbent&GFP at 25 °C. (**a**) PAM-LiCl&GFP, 30%RH; (**b**) AlFum-LiCl&GFP, 30%RH; (**c**) PAM-LiCl&GFP, 60%RH; (**d**) AlFum-LiCl&GFP, 60%RH.

The water sorption rate coefficient, K (s^−1^), provides a clearer insight into the dynamic sorption performance of PAM-LiCl&GFP and AlFum-LiCl&GFP. The LDF fitting for both PAM-LiCl&GFP and AlFum-LiCl&GFP was conducted at 25 °C, 30% RH, and 60% RH, as depicted in Figure 11. The correlation coefficients are consolidated in Table 3. The K for both materials falls within the same order of magnitude, between 8.56 × 10^−4^ and 15.32 × 10^−4^. R^2^ values exceeding 0.99 suggest a strong fit. The water sorption rate coefficients K for PAM-LiCl&GFP are consistently higher than those of AlFum-LiCl&GFP. Specifically, values are 15.32 × 10^−4^ compared to 11.09 × 10^−4^ at 30% RH and 13.03 × 10^−4^ compared to 8.56 × 10^−4^ at 60% RH. Considering this result and previous findings, it is evident that PAM-LiCl&GFP surpasses AlFum-LiCl&GFP in both equilibrium water uptake and sorption rate.

### 3.3. Desorption Performance Analysis

**Figure 12:**
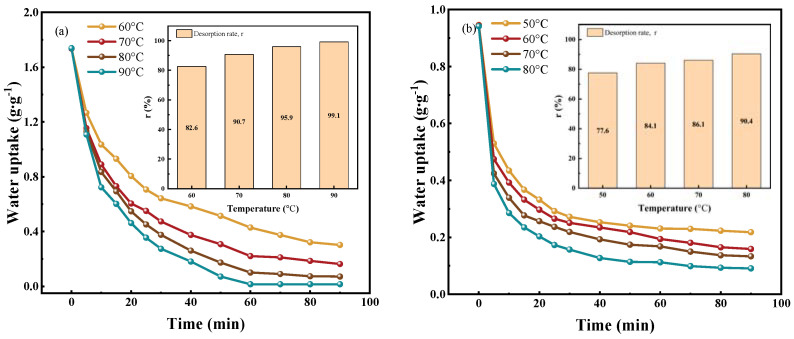
Desorption performance of adsorbent&GFP at different temperatures. (**a**) PAM-LiCl&GFP, (**b**) AlFum-LiCl&GFP.

Beyond water sorption capacity, regeneration performance is vital since it accounts for most of the energy consumption in the rotary dehumidification system. The temperature during regeneration significantly impacts energy consumption. Consequently, we tested the desorption performance of adsorbent&GFP at varied temperatures post achieving water sorption saturation at 25 °C, 60% RH. Figure 12a showcases the dynamic desorption trends of PAM-LiCl&GFP, emphasizing that elevated desorption temperatures result in swifter desorption rates. Despite the DSC analysis (Section 3.1.2) pinpointing a peak desorption rate for PAM-LiCl&GFP at 78 °C, we observed desorption ratios of 82.6% at 60 °C, 90.7% at 70 °C, 95.9% at 80 °C, and 99.1% at 90 °C—all after a span of 90 min. Conversely, PAM-LiCl&GFP’s mass showed minimal variation after 60 min across the working ambient conditions of 70 °C, 80 °C, and 90 °C, signifying the completion of the regeneration process. Figure 12b displays AlFum-LiCl&GFP’s dynamic desorption curve, which attained an 84.1% desorption ratio post 90 min at 60 °C. Yet, AlFum-LiCl&GFP’s initial water uptake, prior to desorption, stands considerably below PAM-LiCl&GFP’s (0.94 g·g^−1^ versus 1.73 g·g^−1^). This suggests that at these or lower RH conditions, a desiccant wheel powered by PAM-LiCl&GFP can deliver superior dehumidification performance with roughly equivalent energy input.

### 3.4. Multiple Dehumidification–Desorption Cycles

**Figure 13:**
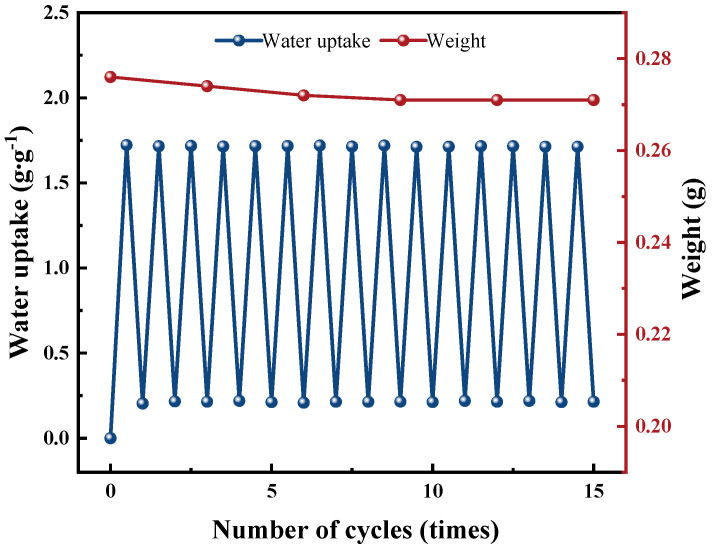
Cycling test of PAM-LiCl&GFP.

The cyclic stability of materials holds significant practical relevance in engineering applications, especially for rotary dehumidification systems. Particularly with a desiccant infused with hygroscopic salts, concerns of shedding from the desiccant wheel substrate (either GFP or CFP) after successive cycles and potential salt leakage arise. In the optimization phase, we fine-tuned the content of each component in PAM-LiCl&GFP to mitigate salt leakage in most operational conditions. Following this, we conducted 15 cycles of water sorption and desorption to assess the loading impact and durability, as illustrated in Figure 13. Each cycle entailed an hour of sorption at 25 °C and 60% RH, succeeded by an hour of desorption at 70 °C. This method ensured near complete saturation during water sorption and thorough desorption of the material. The water sorption process, following the first cycle, commences at approximately 0.2 g·g^−1^ of *w*_a_ because we adopted a drying strategy aligned more with real-world applications rather than fully drying the material, thereby conserving energy. PAM-LiCl&GFP’s water sorption performance evidenced a slight decline over the cycles. The water uptake during the final cycle was 1.712 g·g^−1^, just 0.01 g·g^−1^ below the inaugural cycle. Furthermore, PAM-LiCl&GFP’s mass diminished from 0.276 g to 0.271 g across the 15 cycles, reflecting a mass reduction of roughly 1.8%, with the bulk of the loss occurring within the initial 10 cycles. These findings imply that PAM functions both as a vessel for the hygroscopic salt, LiCl, and as a potent binder, safeguarding against the detachment of the adsorbent material. Collectively, these data underscore the cyclic stability of PAM-LiCl&GFP.

### 3.5. Performance Analysis of Desiccant Wheel Modules

**Figure 14:**
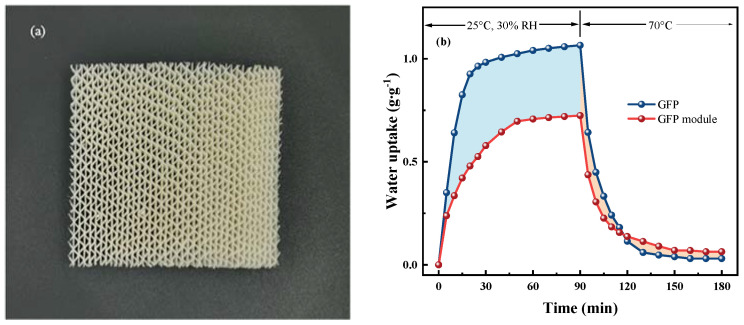
(**a**) Photo of PAM-LiCl&GFP modules, (**b**) Performance of PAM-LiCl&GFP modules.

After optimization of the performance of PAM-LiCl&GFP, PAM-LiCl was integrated into an empty desiccant wheel module without any adsorbent. The module was constructed from GFP with a wavelength of 3.5 mm and a wave height of 1.6 mm, as shown in Figure 14a. The unloaded module measures 50 × 50 × 15 mm and weighs 4.51 g. After the adsorbent introduction, the weight of the desiccant module rose to 10.73 g, with the adsorbent weighing 6.22 g, making up 57% of the total mass. The loaded amount is around 20% less than that of the GFP. This difference stems from the persistent use of the impregnation method, rather than producing PAM-LiCl&GFP first and then combine it with the desiccant wheel module. Tests for water sorption performance at 25 °C, 30% RH, and desorption at 70 °C were carried out on the PAM-LiCl&GFP modules. The results can be seen in Figure 14b. Due to the augmented weight of the desiccant wheel module, the water sorption rate dropped from 11.77 mg·g^−1^∙min^−1^ to 8.05 mg·g^−1^∙min^−1^. Furthermore, the GFP module showed an 81% desorption rate in 30 min at 70 °C. One primary reason for the reduced water sorption and desorption rates is the marginally increased mass transfer resistance in the GFP module. In real-world settings, a fan-induced forced convection would circulate across the desiccant wheel, in contrast to the existing arrangement where it remains in a constant-temperature and -humidity chamber. It is also important to note that the optimized content of PAM-LiCl loading was not found to plug the holes in the desiccant wheel module. In conclusion, the GFP modules crafted with PAM-LiCl maintain a satisfactory performance.

## 4. Conclusions

In the present investigation, we synthesized a composite integrating polyacrylamide (PAM) with glass fiber paper (GFP), a standard substrate for desiccant wheels, augmented with the incorporation of the hygroscopic salt LiCl to bolster its water absorption efficacy. Through physical characterization and experimental analyses, we assessed parameters, including water uptake, water sorption rate, desorption kinetics, and cycling stability, and compared the performance metrics with those of the aluminum fumarate composite GFP. Under conditions characterized by 25 °C and a relative humidity (RH) of 30%, the PAM-LiCl&GFP composite manifested a remarkable water uptake of 1.06 g·g^−1^, notably, exceeding that of AlFum-LiCl&GFP (0.28 g·g^−1^) and silica gel&GFP (0.03 g·g^−1^). In conditions set at 25 °C with a RH of 60%, the PAM-LiCl&GFP composite demonstrated a water uptake of 1.73 g·g^−1^, which stands superior to the 0.94 g·g^−1^ exhibited by AlFum-LiCl&GFP. Furthermore, under scrutiny, the optimized PAM-LiCl&GFP evidenced zero salt efflux at an RH up to 80%, corresponding to a water uptake of 2.84 g·g^−1^. The application of the linear driving force model fitting establishes that PAM-LiCl&GFP exhibits a pronounced superiority in sorption rate when juxtaposed against AlFum-LiCl&GFP. Significantly, the PAM-LiCl&GFP composite realized a laudable desorption ratio at a temperature of merely 60 °C, attaining desorption efficiencies of 82.6%, and a further elevated 90.7% upon elevation to 70 °C. Analyses conducted on the desiccant wheel module, fabricated from PAM-LiCl, elucidated a water uptake of 0.72 g·g^−1^ at ambient conditions of 25 °C and 30% RH. It is also pivotal to note the desiccant wheel modules with no occlusions or clogging, using content of adsorbent components after careful optimization. The presented findings underscore the superiority of PAM-LiCl over AlFum-LiCl in desiccant wheels and also illustrate the possible application of PAM-LiCl in rotary dehumidification.

## Figures and Tables

**Table 1:** Mass of each component of different PAM-LiCl&GFP samples.

	GFP + PAM (g)	+10% LiCl (g)	+15% LiCl (g)	+20% LiCl (g)	+25% LiCl (g)	+30% LiCl (g)
GFP	0.063 ± 0.0001	/	/	/	/	/
GFP + 2%PAM	0.0743 ± 0.0008	0.141	0.178	0.232	0.259	0.307
GFP + 4%PAM	0.0864 ± 0.0014	0.155	0.194	0.241	0.279	0.336
GFP + 6%PAM	0.1237 ± 0.0100	0.243	0.276	0.387	0.435	0.485

**Table 2:** Water uptake *w* (g·g^−1^) at 25 °C for both pristine and metal-loaded materials.

	RH	10%	20%	30%	40%	50%	60%	70%	80%	90%
Samples										
PAM&GFP		0.00	0.00	0.01	0.02	0.05	0.09	0.14	0.19	0.31
AlFum-LiCl&GFP		0.01	0.10	0.28	0.37	0.61	0.94	1.27	1.36	1.42
PAM-LiCl&GFP		0.19	0.76	1.06	1.20	1.44	1.73	2.17	2.84	4.03

**Table 3:** Rate coefficients K and the square of correlation coefficients R^2^ for absorbent&GFP.

	Parameters	25 °C, 30%RH		25 °C, 60%RH	
Sample		K (s^−1^)	R^2^	K (s^−1^)	R^2^
AlFum-LiCl&GFP		11.09 × 10^−4^	0.990	8.56 × 10^−4^	0.991
PAM-LiCl&GFP		15.32 × 10^−4^	0.995	13.03 × 10^−4^	0.999

## Data Availability

Data available on request due to restrictions e.g., privacy or ethical. The data presented in this study are available on request from the corresponding author. The data are not publicly available due to Privacy Policy of the Beijing University of Technology.

## Nomenclature

K	Rate coefficients, s^−1^
*m*_a_	Initial mass of adsorbents, g
*m*_b_	GFP mass after loading PAM, g
*m*_c_	PAM&GFP mass after loading LiCl, g
*m*_e_	Equilibrium mass of adsorbents, g
*r*	Desorption ratio, %
*t*	Time, s
*w*	Water uptake, g·g^−1^
*w*_a_	Initial water uptake, g·g^−1^
*w*_eq_	Equilibrium water uptake, g·g^−1^
*w*_t_	Dynamic water uptake, g·g^−1^
R^2^	Square of correlation coefficients
